# Consciousness—a system-theoretical approach

**DOI:** 10.3389/fpsyg.2014.00115

**Published:** 2014-02-19

**Authors:** Wacław Petryński

**Affiliations:** Department of Tourism and Recreation, Katowice School of EconomicsKatowice, Poland

**Keywords:** consciousness, psychology, motor behavior, human cognition, neuroscience

“Human development has two stages: in the first we think about things; in the second we begin to think about thinking.”

Spiro Agnew

The aim of this work is not to compete with the paper by Boly et al. (2013), but rather to look at the issue of consciousness from a different, system-theoretical perspective. According to advice by Sokal and Bricmont that “*it's a good idea to know, what one is talking about*” (Sokal and Bricmont, 1999, p. 185), let us assume that consciousness is a dynamically changing part of quasi-static whole knowledge of an individual, activated by perception and directed by attention, aimed at dealing with the task just being solved.

Accordingly, while taking as a criterion the scope of field of information, the whole body of an individual's knowledge may be regarded as a **potential consciousness**. It may be divided into two parts: stimulated by attention **active consciousness** and remaining beyond its limits, “sleeping” **inactive consciousness**. The “fuzzy” region between them makes a space for **half-active consciousness**, commonly termed “sub-consciousness.” In this region the access to needed information is not immediate, as in active consciousness, but easier than that in the field of inactive consciousness.

The other dimension of division—independent of the former—results from the fact that knowledge is a mental representation of reality which may be described with various codes. The organic “device” dealing with this issue is the brain, so the knowledge of its structure and evolutionary history may significantly facilitate identification of such codes. Here instructive may be the commonly known division into extrapyramidal and pyramidal systems. The former may be—roughly—associated with sensory experiences (stimuli), and thus it may underlie what may be termed “**real consciousness**.” The latter deals with the abstract representations of reality, stored and processed in one's own memory, so it may be termed “**virtual consciousness**.”

The presented division may be roughly associated with Cartesian division into sensory-mental *res extensa* and purely mental *res cogitans* (Schmaltz, 2008, p. 42), or Pavlov's first and second system of signals (Pavlov, 1973, p. 443). A more detailed division may be traced in the papers by Carpenter (1852), Hughlings Jackson (1884). The latter inspired N.A. Bernstein, who authored probably the most advanced systemic division of information processing in humans, based on evolutionary, and neurophysiological data (Bernstein, 1947). Unfortunately, though Bernstein spoke eight languages, he wrote mainly in Russian, hence even nowadays his works are not very popular in contemporary science, where English prevails.

Bernstein followed the evolutionary development of sense organs, nervous systems, information processing abilities, and motor abilities of living beings. He discerned five levels of movements' construction, tightly joined with specific structures in the central nervous system (CNS), which subsequently appeared in living organisms in the course of evolution:

- A-level, rubro-spinal, responsible for muscle tonus,
- B-level, thalamo-pallidal, responsible for muscle synergies,
- C-level, cortical, pyramidal and striatal, responsible for movements in space,
- D-level, cortical, parietal-premotor, responsible for true representation of reality, embedded in actual spatial and temporal constraints,
- E-level, cortical; Bernstein described it as “***group*** *E, lying over operation level*” (Bernstein, 1947), responsible for fantastic representation of reality, free from actual spatial and temporal constraints.

By the way: Bernstein termed muscle tonus “background of all backgrounds,” i.e., a physiological phenomenon underlying all the motor actions, especially in vertebrates. Analogously, the consciousness makes the mental phenomenon fundamental to any voluntary motor operation as seen from the psychological perspective.

**Figure 1:**
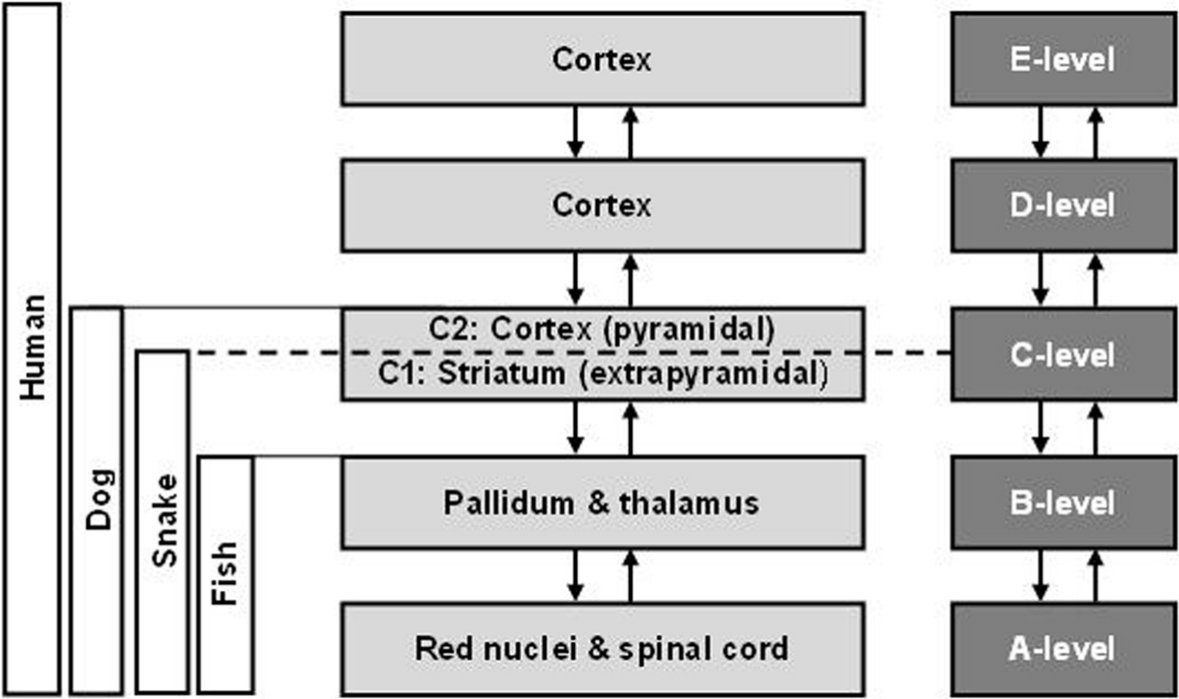
**The system of movements' control levels according to N.A. Bernstein**.

The graphical presentation of Bernstein five-level movements' construction system has been presented in Figure 1 (Petryński, 2008, p. 166).

Unfortunately, Bernstein's theory in its “pure” form is too complex to be directly applicable in practice. To achieve the practical usefulness, it has to be simplified. So, based on Bernstein's evolutionary and neurophysiological theory, it is possible to develop an analogous mental structure including the following modalities of information processing:

- A-level—intrinsic stimuli,
- B-level—extrinsic contact stimuli,
- C-level—extrinsic remote stimuli,
- D-level—verbal code,
- E-level—symbolic code (Petryński and Feigenberg, 2011).

It is worth noticing that in such a “modalities' ladder” (ML) A, B and C levels are “driven” by physical stimuli, whereas the D and E levels are “powered” by purely mental representations of environment, where any connection with reality is possible, indeed, but not necessary. The “common denominator” of Bernstein's model and ML is the same, systemic structure. The final product of the information processing system is a movement, even if merely that of jaws and tongue.

Symptomatically enough, also Rochat (who did not refer to Bernstein) developed a five-level model of self-awareness (Rochat, 2003). Though it is not identical, in general it conforms with ML.

Such differentiation of information processing modalities assigned to particular levels remains in keeping with the scales' conformity premise by Morawski (2005, p. 162). It states that each level of a system has its own information processing modality, as well as temporal and energetic scales of physical phenomena. Accordingly, just the information processing modality makes—to great extent—a specific identity of a given level.

In physics one of the especially important laws is the “correspondence rule” formulated by Jammer (1966). It states—roughly—that at the border region of quantum and classical physics the laws that determine the run of events coincide with each other. Analogously, in the ML there are four such “border regions,” hardly liable to experimental research, yet especially promising scientifically: A–B, B–C, C–D and D–E. Moreover, only the modalities from adjacent levels are to some extent mutually translatable. For example, it is not possible to explain verbally (D-level) how to hold (B-level) an egg strong enough to prevent it from falling down, but not to crush its shell. So, the psychology and motor control are more complex than e.g., physics, what remains in keeping with the classification developed already in 19th century by Comte (2012, pp. 152–154). Summing up, while seen from system-theoretical perspective, the ML makes a specific “skeleton” of consciousness that enables most efficient solving a current task. Even if it includes only mental diagnosis of a given situation in environment.
